# Laboratory Experiments on Anesthesia

**Published:** 1919-01

**Authors:** 


### *Laboratory Experiments to Determine*

I. *The Value of Warmed Oxygenated Ether Vapor compared with Air-Ether Vapor.*

II. *The Value of Pre-Anesthetic Administration of Bicarbonate of Soda in Preventing Acidosis.*

III. *The Value of the Combination of These two Agents.*

By J. T. Gwathmey and E. S. Tibbits. A preliminary note of an article to appear elsewere.

The animal was placed under a glass bell jar in which the air or oxygen was forced, at a fixed pressure (32 mm. Hg.) through a bottle containing ether. The percentage of ether in the mixture passing to the bell jar was constant. The temperature could be regulated. The time was taken from the commencement of the administration of the ether vapors to the stoppage of respiration. The animal was then resuscitated and re-anesthetized 24 or 48 hours later, but in the opposite way, (if the first anesthetic was air-ether, the second was warmed oxygen-ether).

The final results showed that warmed oxygen-ether was more than 2 1/2 times as safe as air-ether. Two of the warmed oxygenated-ether animals were breathing regularly at the end of 22 minutes. One failed to respond to resuscitation, and this was an air-ether experiment. The percentages mentioned above do not express the very great advantage as regards life with warmed oxygenated ether as against air-ether at room temperature.

To determine the value of bicarbonate of soda in preventing acidosis, guinea-pigs were given a hypodermic of 5 cc. of a 3 o/o solution of $NaHCO_3$; they were fed 5 cc. of this agent in milk.

mornings and afternoons. The animals were anesthetized in the middle of the day for 6 days and on the 6th day, while they were still anesthetized, 2 cc. of blood was withdrawn from the heart, and the same amount of physiological salt solution injected.

The experiments showed that when no $NaHCO_3$ was given, the alkaline loss was approximately the same for each anesthetic. When the preliminary $NaHCO_3$ was given, the pigs given the warmed oxygen-ether mixture showed a much higher blood alkalinity than those given the air-ether mixture.

Finally, as between the two agents used, the previous administration of $NaHCO_3$ is of even more importance than the selection of the anesthetic, as far as acidosis is concerned.

